# A comparative study on the cleaning efficacy of a pulsed vacuum cleaning and disinfection device on rigid endoscopic instruments in a hospital setting

**DOI:** 10.3389/fcimb.2025.1607905

**Published:** 2025-08-15

**Authors:** Licong Bo, Xue Wang, Jian Li, Yue Hu

**Affiliations:** ^1^ Supply Room, Shijiazhuang Maternal and Child Health Hospital, Shijiazhuang, Hebei, China; ^2^ Operating Room, Shijiazhuang Maternal and Child Health Hospital, Shijiazhuang, Hebei, China

**Keywords:** pulsed vacuum cleaning and disinfection device, rigid endoscopic instruments, decontamination, instrument damage, protein residue

## Abstract

**Objective:**

Given the increasing demand for rapid and reliable instrument reprocessing to support surgical schedules and minimize infection risks, this study aims to explore the cleaning efficacy of a pulsed vacuum cleaning and disinfection device on rigid endoscopic instruments in a comparative hospital setting.

**Methods:**

A total of 800 rigid endoscopic instruments scheduled for post-operative cleaning in our hospital’s sterilization supply room between July and December 2024 were included in the study. After pre-treatment, the instruments were divided into two groups, with 400 instruments in each group. The control group used a vacuum ultrasonic cleaner, while the observation group used a pulsed vacuum cleaning and disinfection device. The cleaning time, cleaning effect, protein residue detection, and instrument damage rate were compared between the two groups. A cost-effectiveness analysis was also performed.

**Results:**

The cleaning time in the observation group was significantly shorter than that in the control group, with a statistically significant difference (P<0.05). There was no statistically significant difference between the two groups in terms of visual inspection, magnifying light source, and ATP fluorescence comparison (P>0.05). The protein residue detection in the observation group was lower than that in the control group, with a statistically significant difference (P<0.05). The instrument damage rate in the observation group was lower than that in the control group, with a statistically significant difference (P<0.05). The pulsed vacuum device demonstrated significant cost savings, with a lower total cost ($15,984 vs. $21,832) and cost per qualified instrument ($40.26 vs. $55.98) over 400 cycles.

**Conclusion:**

The pulsed vacuum cleaning and disinfection device can effectively clean rigid endoscopic instruments and is worthy of clinical promotion as it enhances operational efficiency and upholds high standards of patient safety by ensuring instrument cleanliness.

## 1 Introduction

The sterilization supply room serves as an indispensable hospital department responsible for cleaning, disinfecting, and sterilizing medical instruments used in clinical operations, thereby ensuring a sterile healthcare environment where the quality of disinfection and sterilization directly impacts medical service functionality, hospital standards, and the incidence of hospital-acquired infections and adverse events (Protano et al., 2019; Hu et al., 2024). Infections linked to inadequately reprocessed endoscopic instruments represent a significant patient safety concern, with global outbreaks of multidrug-resistant organisms underscoring the critical need for highly effective reprocessing methods. Recent evidence reviews demonstrate that persistent contamination on patient-ready endoscopes remains a critical issue, with outbreaks frequently linked to reprocessing failures despite adherence to standard protocols, as highlighted by studies showing ongoing pathogen transmission events even after design improvements and staff training enhancements (Benowitz et al., 2020; Casini et al., 2023). Structural vulnerabilities in complex endoscope designs—such as narrow lumens, internal microdamage, and multi-channel configurations—allow them to harbor biofilms that are highly resistant to standard decontamination and serve as reservoirs for pathogen transmission, a phenomenon corroborated by microbiological surveillance confirming biofilm persistence post-reprocessing under international guidelines (Casini et al., 2023; Kwakman et al., 2023). Recent studies systematically document these patterns, revealing that duodenoscope contamination rates remain problematic even with enhanced protocols, as outbreaks of AmpC-producing E. coli and CRE infections occurred when recommended reprocessing proved insufficient (Scholz et al., 2023) while emerging sterilization technologies like peracetic acid chemical sterilization show promise but require validation against stringent sterility assurance levels (SAL of 10^-6^) to prevent cross-contamination (Rutala and Weber, 2016). The global crisis of multidrug-resistant organisms (MDROs) amplifies these risks, demanding a shift towards next-generation reprocessing technologies capable of overcoming biofilm resilience (Rotello, 2023). This highlights an urgent need for mandatory outbreak reporting, redesigned endoscopes, and the adoption of validated reprocessing protocols, with recent studies continuing to reveal inconsistencies in practices worldwide (Al Mayahi et al., 2019; Ofstead et al., 2022).

In recent years, China’s healthcare industry has expanded significantly, with a large-scale increase in the use of minimally invasive instruments, rigid endoscopic devices, and dental surgical instruments. Consequently, the volume of instruments processed in sterilization supply rooms has risen, which demands higher cleaning and disinfection standards (Liu et al., 2021). Rigid endoscopic instruments, in particular, pose greater challenges due to their complex internal structures, high precision, and special materials. During surgery, blood and other contaminants are often retained within the instrument, making them more difficult to clean than standard surgical tools (Pereira et al., 2015). If not thoroughly cleaned, these residues can form biofilms that hinder the penetration of sterilizing agents, thereby affecting the sterilization effectiveness and increasing the risk of infection (Wang et al., 2023). This entire process, from point-of-use pre-treatment to final sterilization, relies on clear protocols and diligent execution by healthcare staff, highlighting the importance of interprofessional collaboration between operating room and sterilization department teams to address operational inefficiencies such as staffing time mismatches (Hu et al., 2023). The active participation and competence of staff are fundamental to achieving optimal outcomes, as evidenced by interventions that improve timely cleaning practices and reduce biofilm formation risks (Hu et al., 2023).

Traditional cleaning methods for rigid endoscopic instruments typically involve flushing techniques. However, cleaning machines that interface with these devices face limitations in cleaning capacity and quality, as the design restricts the number of instruments processed per cycle. This method no longer meets the demands of modern hospitals for the cleaning of such specialized instruments (Choi et al., 2021). Furthermore, while spray cleaning machines are used, their structural limitations prevent thorough cleaning of rigid endoscopes. Although redesigned flow interfaces have been attempted to address these shortcomings, the cleaning results often fall short of expectations, and the loading time and capacity for cleaning instruments remain constrained (Anderson et al., 2023).

Ultrasonic cleaning machines, traditionally used for instrument cleaning, also present challenges. These devices cannot perform disinfection and drying simultaneously, nor can they effectively flush out contaminants after cleaning, leading to suboptimal cleaning outcomes due to their inability to remove microscopic biofilm formations consistently (Mirzaei et al., 2020).

In response to these challenges, the pulsed vacuum cleaning and disinfection device was developed. This device integrates cleaning, rinsing, disinfection, and drying functions into one system, offering a high degree of automation. It has been found to perform well in cleaning minimally invasive instruments and dental surgical tools. The device can accommodate instruments in a variety of loading configurations and quantities without strict requirements, allowing instruments to be placed freely in the cleaning baskets for processing (Chang and Tao, 2019). This underscores the need for robust training and advanced clinical reasoning among nursing staff, who are pivotal in implementing and overseeing these critical processes (Wilder and Roth, 2012). The evolution towards more complex surgical instruments necessitates not only technological advancements in cleaning but also a parallel development in the roles and competencies of healthcare professionals, including advanced practice nurses and other specialized non-medical staff within the surgical and sterilization workflow (Lowe et al., 2012).

Although pulsed vacuum cleaning and disinfection devices have gradually gained popularity in domestic sterilization supply centers, there remains limited research on their application. This study aims to investigate the cleaning effectiveness of the pulsed vacuum cleaning and disinfection device on rigid endoscopic instruments.

## 2 Materials and methods

This study was a prospective, non-randomized comparative trial. This manuscript has been prepared in accordance with the Transparent Reporting of Evaluations with Nonrandomized Designs (TREND) statement checklist (**Supplementary File 1**) (Des Jarlais et al., 2004).

### 2.1 General information

A total of 800 rigid endoscopic instruments scheduled for post-operative processing in the sterilization supply room of our hospital between July and December 2024 were included in the study. After pre-treatment, the instruments were divided into two groups based on the cleaning method: the control group and the observation group, with 400 instruments in each group.

#### 2.1.1 Observation group

122 laparoscopes, 97 hysteroscopes, 65 arthroscopes, 47 nephroscopes, 43 prostate resection scopes, 26 thoracoscopes;

#### 2.1.2 Pollution level

265 items with mild contamination, 81 items with moderate contamination, and 54 items with severe contamination;

#### 2.1.3 Usage frequency

102 items used ≤1 time/month, 229 items used 2–5 times/month, and 69 items used ≥6 times/month.

#### 2.1.4 Control group

131 laparoscopes, 94 hysteroscopes, 71 arthroscopes, 39 nephroscopes, 43 prostate resection scopes, 22 thoracoscopes;

#### 2.1.5 Pollution level

269 items with mild contamination, 83 items with moderate contamination, and 48 items with severe contamination;

#### 2.1.6 Usage frequency

105 items used ≤1 time/month, 222 items used 2–5 times/month, and 73 items used ≥6 times/month.

There was no significant difference in the types of instruments, contamination levels, or usage frequency between the two groups (P>0.05).

Both groups of rigid endoscopic instruments were managed by the same batch of staff in the sterilization supply center, with 11 female members in total. The age range of staff was 25-40 years, with an average age of (32.85±5.45) years, and work experience ranging from 3 to 15 years, with an average of (10.09±2.87) years. The staff's education levels were as follows: 2 had a technical secondary school diploma, and 9 had a bachelor's degree.

### 2.2 Methods

#### 2.2.1 Cleaning tools

The cleaning equipment used in this study included devices produced by Shandong Xinhua Medical, including a pulsed vacuum cleaning machine (Model PC-150L), high-efficiency fully automatic cleaning and disinfection machine (Model super-6000), spray cleaning rack (Model super6000-05E-X), medical drying cabinet (Model YGZ-1600S), and pressure water gun (Model Center-R5).

#### 2.2.2 Detection tools

Detection tools included magnifying glasses with light sources, An Yika protein residue detection device (Model MINIPRO), and test rods (Model PROMICO).

#### 2.2.3 Control group cleaning process using ultrasonic cleaner

##### 2.2.3.1 Manual pre-treatment

The pre-treatment process begins at the point of use in the operating room. Immediately after the surgical procedure, trained personnel wipe external debris from the instruments and flush the internal channels with sterile water to prevent the drying of bioburden and biofilm formation. This initial step is critical for effective downstream processing. After surgery, the rigid endoscopic instruments used during the operation were then placed in designated sealed transport containers and uniformly collected and transported to the hospital’s sterilization supply center. The instrument labels were carefully checked to confirm the correct quantity, and the appearance and functionality of the instruments were inspected. The instruments were categorized and placed in cleaning baskets. The instruments were removed from the baskets, and manually disassembled to the smallest units. Under running water, residual blood and stains were rinsed off. If dried blood could not be removed, the instruments were soaked in a 30-40°C multi-enzyme cleaning solution. Subsequently, a high-strength water gun was used for rinsing.

##### 2.2.3.2 Ultrasonic cleaning

The ultrasonic cleaning machine was embedded in the endoscope cleaning workstation. The first tank was used for ultrasonic cleaning, and multi-enzyme cleaning solution was added to soak the rigid endoscopic instruments. The liquid level needed to be higher than the surface of the instruments. The ultrasonic cleaning program was then activated, with a water temperature set between 30–45°C and the cleaning time set for a minimum of 5 minutes.

##### 2.2.3.3 Spray cleaning and drying

The second tank was used for spray cleaning and drying. The rigid endoscopic instruments were placed in the dedicated baskets and loaded into the spray cleaning and disinfection machine. After closing the loading door, the program was started. High-pressure cold and hot water sprays were used for cleaning, followed by high-temperature drying.

#### 2.2.4 Observation group cleaning process using pulsed vacuum cleaning and disinfection machine

##### 2.2.4.1 Manual pre-treatment

The pre-treatment process was the same as the control group.

##### 2.2.4.2 Post-pre-treatment cleaning

After pre-cleaning, the instruments were loaded onto a dedicated instrument tray and placed in the pulsed vacuum cleaning and disinfection machine. Based on the number of instruments, the appropriate water level was selected. The suitable loading rack was selected, and the machine cleaning program was set, with the temperature set at 93°C and the cleaning time for 2.5 minutes. After cleaning, vacuum drying was performed.

### 2.3 Observation indicators

#### 2.3.1 Cleaning time

The cleaning time for each group of 400 rigid endoscopic instruments was recorded. The time was counted from the start of manual cleaning until all instruments had been cleaned and the time stopped.

#### 2.3.2 Cleaning effect

The cleaning effect was compared between the two groups. The “Hospital Sterilization Supply Center Part 3: Cleaning, Disinfection, and Sterilization Effect Monitoring Standards” (Central sterile supply department (CSSD) - part 3: surveillance standard for cleaning, disinfection and sterilization WS310.3-2016, 2017) was used to evaluate the cleaning quality through visual inspection, magnifying light source observation, and Adenosine Triphosphate (ATP) fluorescence measurement.

#### 2.3.3 Visual inspection

The researcher visually checked whether there were any contaminants on the surface of the rigid endoscopic instruments. For metal aspirators, a special cotton swab was used to wipe the instrument. If no contaminants were found on the instrument surface or the swab showed no color change, the cleaning was considered acceptable. If contaminants were found, the cleaning was considered inadequate.

#### 2.3.4 Magnifying light source observation

The cleaned instruments were examined using a magnifying glass with a light source. If no contaminants (e.g., scale, stains, blood) were visible, the cleaning was considered acceptable; otherwise, it was considered inadequate.

#### 2.3.5 ATP fluorescence detection

Detection rods were used to sample the surface, lumen, and valve of the rigid endoscopic instruments. The relative light units (RLU) were measured after activating the solution. If the RLU value was ≤2000, the cleaning was considered acceptable; if it was higher, the cleaning was considered inadequate.

#### 2.3.6 Protein residue detection

Protein residue was quantified using a protein residue test kit based on the biuret method. For each instrument, sampling was performed by swabbing a standardized 10 cm² surface area of a critical site (a joint or lumen opening). The swab was processed according to the manufacturer’s protocol, and the color change was used to evaluate the protein residue on the instrument surface after a 5-minute incubation period. Green indicated clean, gray indicated mild contamination, light purple indicated moderate contamination, and dark purple indicated severe contamination.

#### 2.3.7 Instrument damage

After cleaning, the instruments were checked for damage, and the number of damaged instruments was recorded.

### 2.4 Statistical analysis

Statistical analysis was performed using SPSS 26.0 software. Normally distributed data were described using means ± standard deviation and analyzed using a t-test. Data that did not follow a normal distribution were described using the median and interquartile range [M (Q1-Q3)] and analyzed using the rank sum test. Count data were described using frequency (proportion) and analyzed using the chi-square test. P<0.05 was considered statistically significant.

## 3 Results

### 3.1 Comparison of cleaning time between groups

**Figure 1:**
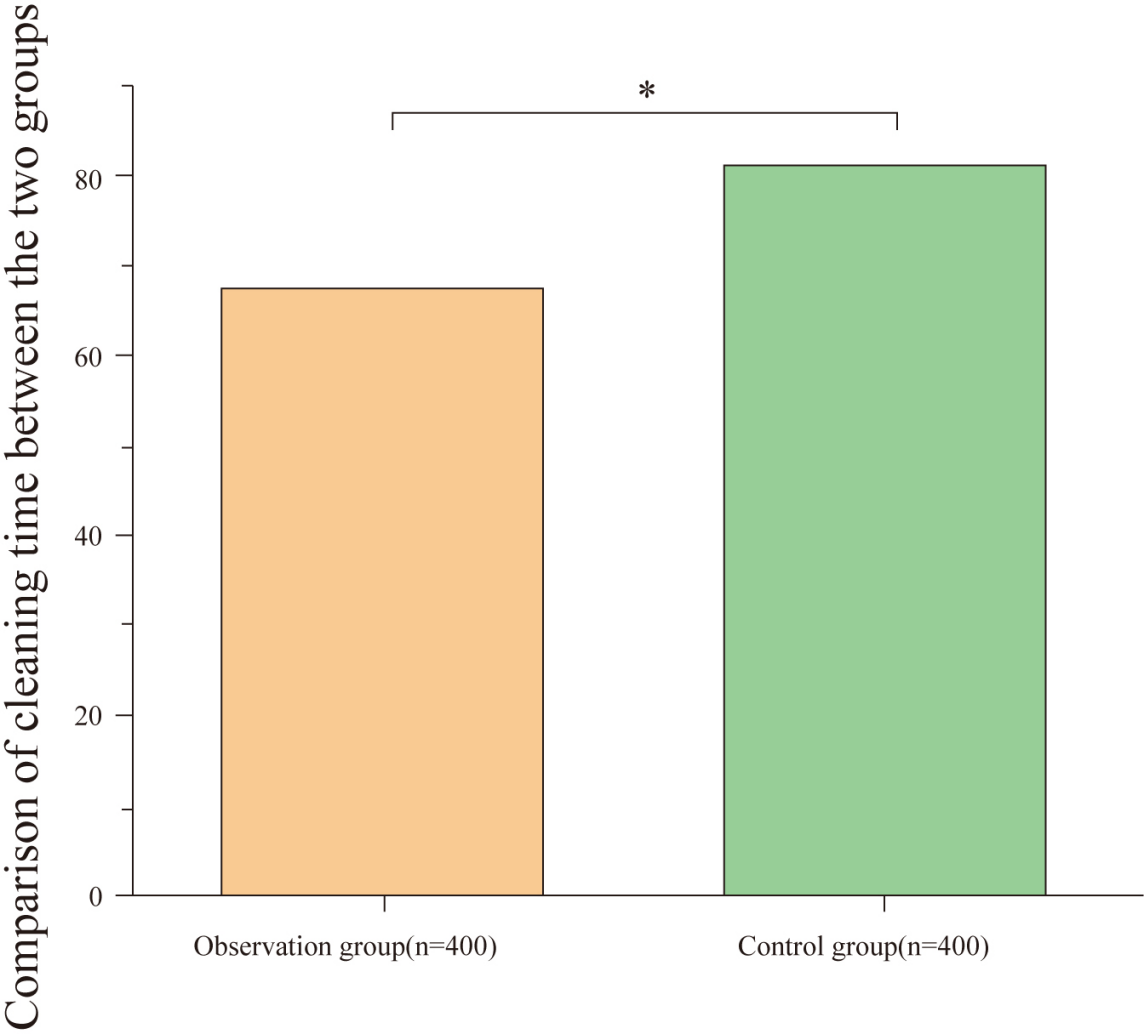
Comparison of cleaning time between groups. The figure displays the average cleaning time for the observation and control groups. The cleaning time in the observation group (67.39 ± 7.32 minutes) was significantly shorter than in the control group (81.24 ± 7.21 minutes), with a statistically significant difference (*P < 0.001).

The cleaning time in the observation group was significantly lower than in the control group, and the difference was statistically significant (P<0.05), as shown in **Table 1** and **Figure 1**.

**Table 1:** Comparison of cleaning time between groups (mean ± standard deviation, min).

Group	Cleaning time (min)	t value	P value
Observation Group (n = 400)	67.39 ± 7.32	26.960	<0.001
Control Group (n = 400)	81.24 ± 7.21		

### 3.2 Comparison of cleaning effect between groups

**Figure 2:**
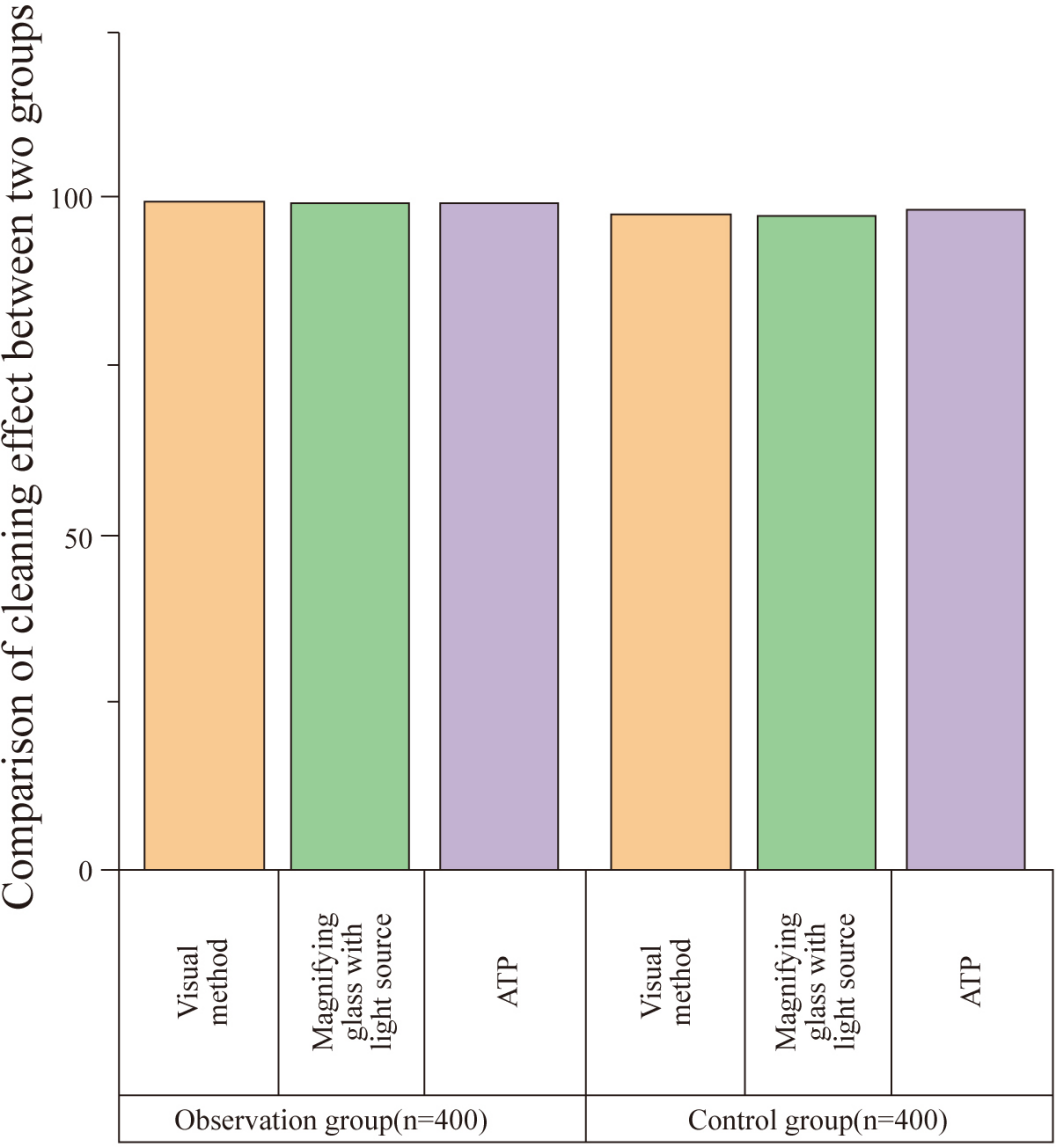
Comparison of cleaning effect between groups. This figure compares the cleaning effect between the observation and control groups as assessed by visual inspection, magnifying light source, and ATP fluorescence detection. (ATP: Adenosine Triphosphate). No statistically significant difference was found between the two groups in any of the assessments (P > 0.05). The qualification rates for visual inspection, magnifying light source observation, and ATP fluorescence detection were 99.50%, 99.25%, and 99.50% for the observation group (n = 400) and 98.00%, 97.50%, and 98.25% for the control group (n = 400), respectively.

There was no statistically significant difference in the cleaning effect between the two groups as assessed by visual inspection, magnifying light source observation, and ATP fluorescence detection (P>0.05). The results are summarized in **Table 2** and **Figure 2**.

**Table 2:** Comparison of cleaning effect between groups (n, %).

Group	Visual Inspection		Magnifying Light Source		ATP Fluorescence Detection	
	Qualified Items	Qualification Rate (%)	Qualified Items	Qualification Rate (%)	Qualified Items	Qualification Rate (%)
Observation Group (n = 400)	398	99.50	397	99.25	398	99.50
Control Group (n = 400)	392	98.00	390	97.50	393	98.25
χ²	3.646		3.831		2.809	
P	0.056		0.050		0.094	

### 3.3 Comparison of protein residue detection between groups

**Figure 3:**
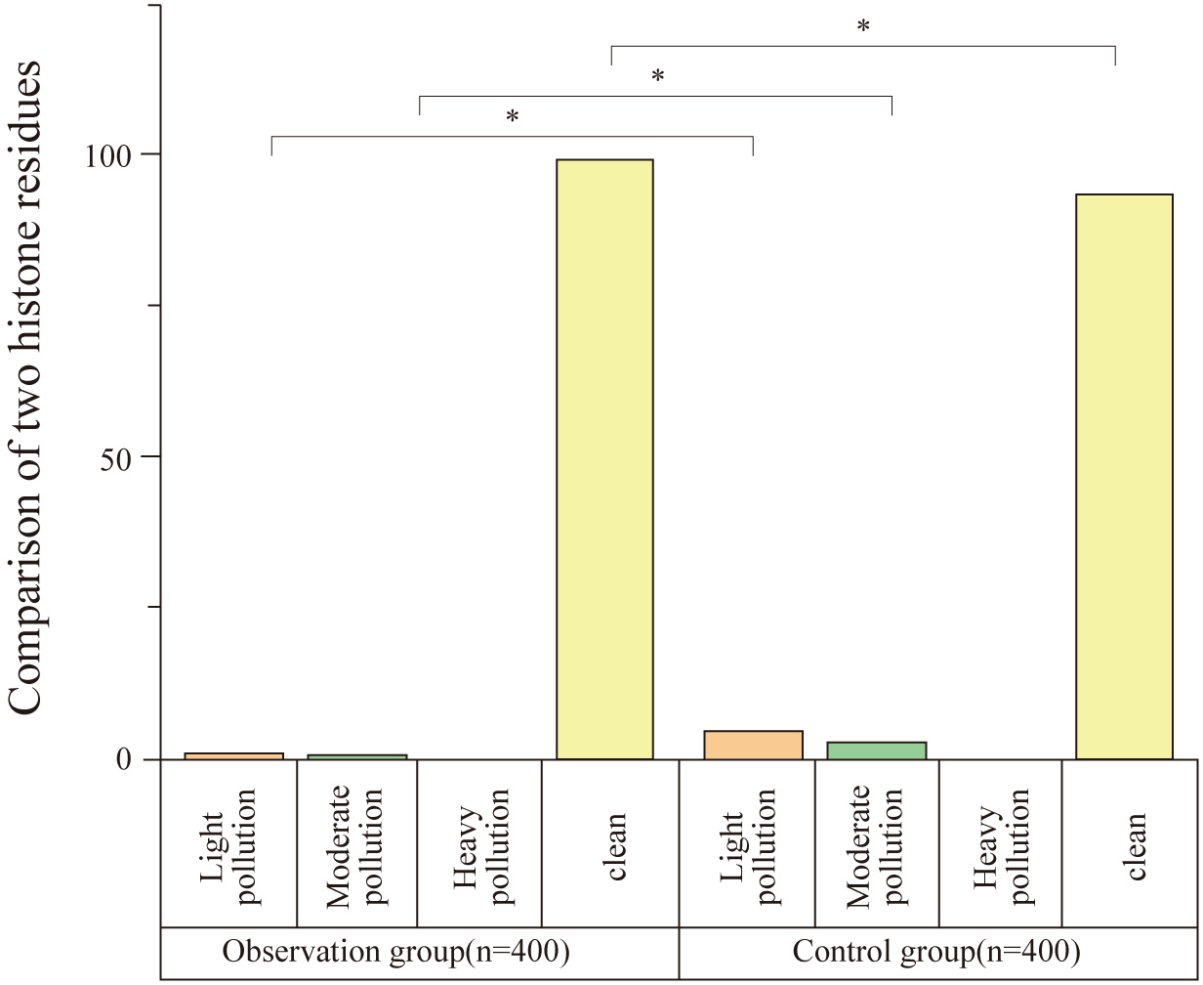
Comparison of protein residue detection between groups. This figure shows the comparison of protein residue levels between the observation and control groups. The observation group had significantly lower levels of protein contamination, with 98.75% of instruments showing no contamination compared to 93.25% in the control group (n = 400). Statistically significant differences were observed in both mild and moderate contamination rates (*P < 0.05).

The protein residue level in the observation group was significantly lower than that in the control group, with a statistically significant difference (P<0.05), as shown in **Table 3** and **Figure 3**.

**Table 3:** Comparison of protein residue detection between groups (n, %).

Group	Mild Contamination n (%)	Moderate Contamination n (%)	Severe Contamination n (%)	Clean Items (No Contamination) n (%)
Observation Group (n = 400)	3 (0.75%)	2 (0.50%)	0	395 (98.75%)
Control Group (n = 400)	18 (4.50%)	9 (2.25%)	0	373 (93.25%)
χ² value	11.003	4.517	–	15.775
P value	0.001	0.034	–	<0.001

### 3.4 Comparison of instrument damage between groups

**Figure 4:**
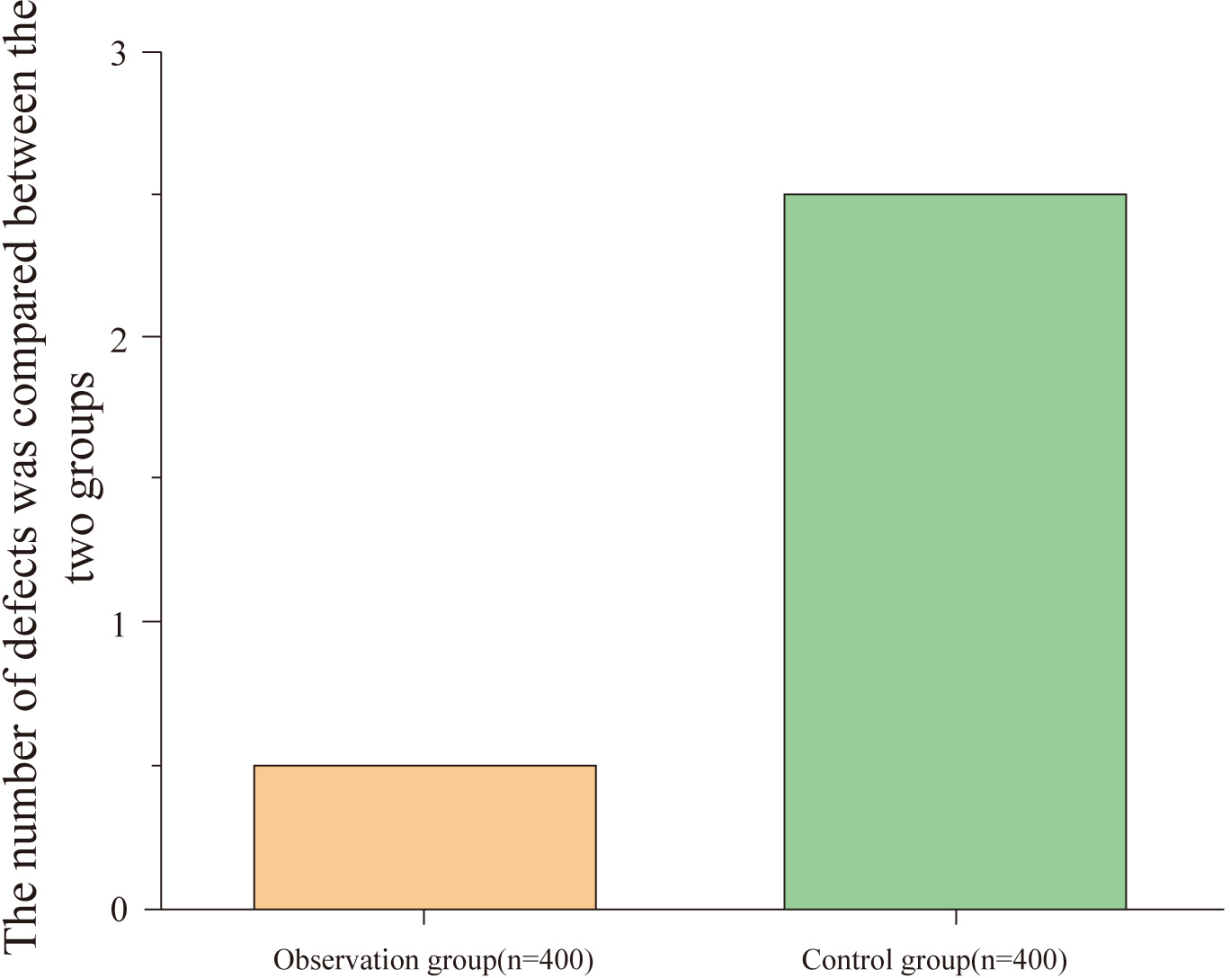
Comparison of instrument damage between groups. This figure compares the instrument damage rates between the observation and control groups. The observation group (n = 400) had a significantly lower instrument damage rate, with fewer instances of damage compared to the control group (n = 400) (P < 0.05).

The observation group had a lower instrument damage rate compared to the control group, with a statistically significant difference (P<0.05), as shown in **Table 4** and **Figure 4**.

**Table 4:** Comparison of instrument damage between groups (n, %).

Group	Damaged Instruments	Damage Rate (%)	χ² value	P value
Observation Group (n = 400)	2	0.500	5.415	0.020
Control Group (n = 400)	10	2.500		

### 3.5 Cost-effectiveness analysis

The estimated cost-effectiveness analysis for 400 cleaning cycles revealed a significant economic advantage for the observation group. The total cost for the observation group was $15,984, compared to $21,832 for the control group. This was primarily driven by lower labor costs ($8,984 vs. $10,832) and substantially lower instrument repair costs ($1,000 vs. $5,000). Consequently, the average cost per qualified instrument was 28.1% lower in the observation group ($40.26) than in the control group ($55.98), as detailed in **Table 5**.

**Table 5:** Estimated cost-effectiveness analysis per 400 cleaning cycles.

Cost Parameter	Observation Group (n = 400)	Control Group (n = 400)
Labor Costs (at $20/hour)	$8,984	$10,832
Consumable Costs (est. $15/cycle)	$6,000	$6,000
Instrument Repair Costs (est. $500/repair)	$1,000	$5,000
Total Estimated Cost	$15,984	$21,832
Total Qualified Instruments (Magnifying Light)	397	390
Cost per Qualified Cycle	$40.26	$55.98

## 4 Discussion

Hospital infection management guidelines (Central sterile supply department (CSSD) - part 3: surveillance standard for cleaning, disinfection and sterilization WS310.3-2016, 2017) indicate that rigid endoscope instruments must be thoroughly cleaned and sterilized after use. If the instruments are not cleaned properly and residual organic material remains, it may obstruct the penetration of sterilizing agents during the disinfection process, resulting in inadequate sterilization. Therefore, cleaning is a prerequisite for the disinfection and sterilization of instruments. Additionally, for some rigid endoscope instruments with complex structures and intricate designs, blood clots, tissue fragments, and other debris are more likely to remain in the hard-to-reach crevices of the instrument, potentially fostering bacterial or spore formation, which ultimately negatively affects the disinfection and sterilization effectiveness (Baboudjian et al., 2023; Mora-Galván et al., 2024). Rutala et al (Valverde-López et al., 2021). pointed out that when used properly, disinfection and sterilization can ensure the safe use of both invasive and non-invasive medical devices. Cleaning should always precede high-level disinfection and sterilization. Strict adherence to current disinfection and sterilization guidelines is critical in preventing patient infections and exposure to infectious pathogens. Relevant studies (Vincenti et al., 2021; Eussen et al., 2024) have shown that the cleaning effectiveness of rigid endoscopes is influenced by several factors, such as the complexity of the instrument’s structure, the degree of contamination, and the cleaning method. Among these, the cleaning method directly impacts the final cleanliness of the instrument, making it essential to improve cleaning methods to enhance cleaning quality. Currently, clinical sterilization supply departments primarily use cleaning machines for instrument cleaning, which can fully meet the cleanliness requirements of the instruments and help prolong their service life. In recent years, ultrasonic cleaning machines have been widely used in clinical sterilization supply departments, with spraying head flushing technology as the main approach (Hofmann et al., 2023; Zhang et al., 2023). However, during the actual cleaning process of rigid endoscope instruments, it is necessary to constantly adjust the cleaning nozzles, and the instruments cannot be directly placed in the cleaning tray, which complicates the cleaning procedure (Liu et al., 2023).

The pulsed vacuum cleaning and disinfection machine is a more efficient cleaning and disinfection device suitable for cleaning various medical instruments, such as general clinical instruments, minimally invasive instruments, and dental surgical instruments. The pulsed vacuum cleaning and disinfection machine combines vacuum pulses with ultrasonic disinfection to achieve the cleaning purpose. It transforms the traditional manual cleaning process, which involves classifying and separating instruments, into an integrated cleaning and disinfection system. This system can automatically complete the full process of pulsed vacuum cleaning, ultrasonic cleaning, thermal disinfection, and vacuum drying (Liu et al., 2023). Liu et al (Liu et al., 2023). pointed out that the pulsed vacuum cleaning and disinfection machine can effectively improve the cleaning quality of dental instruments and reduce cleaning time. Therefore, it should be widely applied and promoted in clinical settings. The results of this study show that the cleaning time in the observation group was significantly shorter than in the control group (P < 0.05), indicating that the pulsed vacuum cleaning and disinfection machine can reduce the cleaning time for rigid endoscope instruments. This may be because the application of the pulsed vacuum cleaning and disinfection machine in sterilization supply departments helps shorten the cleaning time, saving manpower and resources, thus improving the instrument turnover efficiency. The nozzles and hose connections in ultrasonic cleaners are prone to contamination and need regular cleaning, which increases the workload of staff and prolongs the cleaning time (Zhou et al., 2020). In contrast, the pulsed vacuum cleaning and disinfection machine does not have strict limitations regarding the loading method and type of instruments, allowing for a larger loading capacity and greatly shortening cleaning time, thereby improving cleaning efficiency while maintaining cleaning quality (Zhou et al., 2020).

In a study by Zhou et al (Zhou et al., 2020), the pulsed vacuum cleaning and disinfection machine was observed for its effectiveness in cleaning endoscope instruments. Group B, using the pulsed vacuum machine, was compared with Group A, which used an ultrasonic cleaner. The results showed that Group B had a higher cleaning degree and a higher negative rate of Jelly contamination, as well as a lower ATP fluorescence positive rate than the control group (P < 0.05). The findings of this study also revealed that there was no significant difference between the two groups in terms of visual inspection, magnifying light source, and ATP fluorescence (P > 0.05), suggesting that the use of the pulsed vacuum cleaning and disinfection machine on rigid endoscope instruments achieved comparable results to ultrasonic cleaning, consistent with previous studies. This further indicates that the pulsed vacuum cleaning and disinfection machine, when applied in sterilization supply departments for cleaning rigid endoscope instruments, outperforms the ultrasonic cleaner and significantly improves cleaning quality.

The reasons for this can be explained by the pulsed vacuum cleaning and disinfection machine’s design, which includes an automatic walking unit around the outer frame. This design allows the cleaning machine to travel smoothly through internal pipes, enabling rapid cleaning even in deep and curved areas of the instrument. The pulsed water vapor can directly spray into these areas, maximizing the cleaning effect. Additionally, the machine can perform vacuum degassing treatment, which causes the tiny bubbles in the cleaning liquid to expand rapidly as the pressure decreases. This enhances the scrubbing force on the instrument’s surface and accelerates the loosening and removal of biofilms, ultimately improving surface cleanliness (Zhang et al., 2023).

Secker et al (Secker et al., 2020). highlighted that an effective cleaning method could eliminate residual proteins and amyloid protein contaminants, potentially reducing hospital-acquired infections. Yao et al (Zhou et al., 2020). also noted that the pulsed vacuum cleaning and disinfection machine’s application in endoscope cleaning had more significant effects, shortening cleaning time, improving efficiency, reducing Jelly negative rate, ATP fluorescence positive rate, and RLU values, and decreasing protein residue. The results of this study also showed that the observation group had a higher cleaning rate than the control group (P > 0.05), suggesting that the pulsed vacuum cleaning and disinfection machine can improve the cleaning rate of rigid endoscope instruments and reduce protein residue. This may be because the pulsed cleaning and disinfection machine uses a new drying technology that combines jacket-structured vacuum drying with hot air drying, allowing instruments to avoid secondary drying and saving drying time. The working principle and loading basket design of the pulsed cleaning and disinfection machine also enable more instruments to be cleaned and disinfected in a shorter period.

Zhou et al (Zhou et al., 2020). found that the pulsed vacuum cleaning and disinfection machine is effective in cleaning endoscope instruments, reducing cleaning time without increasing instrument damage rate, and thus it is worth promoting in clinical applications. The results of this study showed that the instrument damage rate in the observation group was significantly lower than that in the control group (P < 0.05), indicating that the pulsed vacuum cleaning and disinfection machine can reduce the damage rate of rigid endoscope instruments. When operating, the pulsed vacuum cleaning machine can precisely control pressure changes. During the vacuum phase, the air is gradually extracted from the chamber, preventing sudden pressure changes that could cause strong suction and damage to the instruments. During the injection of cleaning liquids and steam, the pressure increases steadily and stays within the safe range for the instruments, preventing damage from excessive pressure. Furthermore, the machine can accurately regulate the temperature, typically maintaining it between 50°C and 90°C during cleaning and disinfection. This temperature range is effective for cleaning while being lower than the thermal deformation temperature of materials such as metals and plastics used in rigid endoscopes, ensuring that the instruments will not deform or be damaged by excessive heat.

### 4.1 Perspectives for clinical practice and future directions

The findings of this study have significant implications for clinical practice, particularly concerning patient safety and operational efficiency. The adoption of advanced cleaning technologies like the pulsed vacuum washer-disinfector is not merely a technological upgrade but also a catalyst for evolving professional roles and practices within the hospital. The successful implementation of such systems requires a highly skilled workforce. Clinical Nurse Specialists (CNS), for example, can play a pivotal role in overseeing the quality management of instrument reprocessing, developing evidence-based protocols, and providing advanced training to staff, thereby enhancing safety in the operating theater and sterilization department (Nilsen et al., 2020). Furthermore, the complexity of modern surgical instruments demands continuous education and competency validation for all personnel involved. Knowledge and adherence to best practices by operating room and sterilization supply department nurses are fundamental to preventing an effective reprocessing cycle, as deviations can directly contribute to surgical site infections (Heibeyn et al., 2021). However, achieving seamless integration and collaboration between these departments is often challenging. Overcoming barriers such as communication gaps and differing departmental priorities—rooted in organizational fragmentation—is essential for effective interprofessional teamwork, which is a cornerstone of patient safety in the surgical environment, as systematic assessments reveal that these issues, including hierarchical conflicts and resource-related tensions, significantly increase the risk of surgical complications and require multi-level interventions addressing individual, team, and systems factors to ensure sustainable improvements in teamwork and safety outcomes (Etherington et al., 2021; van Dalen et al., 2022). Moreover, the COVID-19 pandemic reinforced the critical importance of impeccable infection control, highlighting the risks associated with aerosol-generating procedures and placing an even greater emphasis on stringent reprocessing protocols to prevent nosocomial transmission (Repici et al., 2020). Future research should focus on the long-term impact of these advanced cleaning systems on instrument longevity, the incidence of surgical site infections, and cost-effectiveness analyses. Additionally, studies exploring the development of standardized educational programs and teamwork-building interventions for reprocessing staff would be highly valuable.

## 5 Limitations

This study has several limitations that should be acknowledged. First, this was a single-center study, which may limit the generalizability of our findings to other healthcare settings with different patient populations, surgical caseloads, or institutional protocols. Second, our assessment of cleaning efficacy focused on physical and chemical indicators (visual inspection, ATP, protein residue) rather than microbiological analysis. While these are standard methods, future studies should integrate microbial cultures (e.g., biofilm PCR assays). Third, as acknowledged by the addition of our preliminary cost-effectiveness data, a more formal and comprehensive economic evaluation is needed to fully assess the financial viability for healthcare institutions with varying capital budgets. Finally, while we monitored for instrument damage, a longer-term follow-up would be beneficial to assess the cumulative impact of each cleaning method on the lifespan of the instruments.

## 6 Conclusion

In conclusion, the pulsed vacuum cleaning and disinfection machine demonstrates superior performance compared to the traditional ultrasonic cleaner for reprocessing rigid endoscopic instruments. It significantly reduces cleaning time, lowers the risk of instrument damage, and is more effective at removing protein residues, all of which contribute to higher operational efficiency and enhanced patient safety. Our preliminary economic analysis suggests these operational gains also translate into significant cost savings, reinforcing the device’s value proposition. While the initial investment may be higher, the long-term benefits in terms of faster instrument turnover and improved cleaning quality make it a highly valuable technology for modern sterilization supply centers. However, to fully leverage the benefits of such advanced systems, healthcare institutions must also invest in robust staff training, foster a culture of interprofessional collaboration, and support the advanced roles of nursing professionals in quality management. The pulsed vacuum cleaning and disinfection machine is highly recommended for clinical promotion as a key component of a comprehensive strategy to ensure the highest standards of instrument decontamination.

## Data Availability

The original contributions presented in the study are included in the article/**Supplementary Material**. Further inquiries can be directed to the corresponding author.
