# Supplementary material for: A comparative study on the cleaning efficacy of a pulsed vacuum cleaning and disinfection device on rigid endoscopic instruments in a hospital setting
Source: Front Cell Infect Microbiol. 2025 Aug 15;15:1607905. doi: 10.3389/fcimb.2025.1607905 (PMC12394517; doi:10.3389/fcimb.2025.1607905)
Supplement: Supplementary file 1 [file DataSheet1.docx]

**Supplementary File 1**

**TREND Statement Checklist**

| **Paper Section/Topic** | **Item No** | **Descriptor** | **Reported?** | **Pg #** |
| --- | --- | --- | --- | --- |
| **Title and Abstract** | | | | |
| Title and Abstract | 1 | Information on how units were allocated to interventions, structured abstract recommended, information on target population or study sample. | ✓ | 1-3 |
| **Introduction** | | | | |
| Background | 2 | Scientific background and explanation of rationale. Theories used in designing behavioral interventions. | ✓ | 3-7 |
| **Methods** | | | | |
| Participants | 3 | Eligibility criteria for participants, method of recruitment, recruitment setting, settings and locations where data were collected. | ✓ | 7-8 |
| Interventions | 4 | Details of the interventions for each study condition, including content, delivery method, unit of delivery, deliverer, setting, exposure quantity, duration, and time span. | ✓ | 8 |
| Objectives | 5 | Specific objectives and hypotheses. | ✓ | 6-7 |
| Outcomes | 6 | Clearly defined primary and secondary outcome measures. Methods used to collect data and enhance quality of measurements. Information on validated instruments. | ✓ | 10-11 |
| Sample Size | 7 | How sample size was determined. | N/A | - |
| Assignment Method | 8 | Unit of assignment, method used to assign units to study conditions, and methods to minimize bias from non-randomization. | ✓ | 7 |
| Blinding (masking) | 9 | Whether participants, those administering interventions, and those assessing outcomes were blinded. | N/A | - |
| Unit of Analysis | 10 | Description of the smallest unit for analysis and any adjustments for differing units of assignment vs. analysis. | ✓ | 8 |
| Statistical Methods | 11 | Statistical methods used for primary and additional analyses, including methods for correlated data, subgroup analyses, missing data, and software used. | ✓ | 12 |
| **Results** | | | | |
| Participant flow | 12 | Flow of participants through each stage of the study. A diagram is strongly recommended. | N/A | - |
| Recruitment | 13 | Dates defining the periods of recruitment and follow-up. | ✓ | 7 |
| Baseline Data | 14 | Baseline demographic and clinical characteristics for each group. | ✓ | 12 |
| Baseline equivalence | 15 | Data on study group equivalence at baseline and statistical methods used. | ✓ | 12 |
| Numbers analyzed | 16 | Number of participants (denominator) included in each analysis for each study condition. | ✓ | 12 |
| Outcomes and estimation | 17 | For each outcome, a summary of results for each study condition, effect size, and confidence interval. | ✓ | 12-13 |
| Ancillary analyses | 18 | Summary of other analyses performed (e.g., subgroup). | N/A | - |
| Adverse events | 19 | Summary of all important adverse events or unintended effects. | ✓ | 13 |
| **Discussion** | | | | |
| Interpretation | 20 | Interpretation of the results, taking into account study hypotheses, sources of potential bias, imprecision, and other limitations. Discussion of mechanisms and implementation success. | ✓ | 13-16 |
| Generalizability | 21 | Generalizability (external validity) of the trial findings. | ✓ | 16-18 |
| Overall Evidence | 22 | General interpretation of the results in the context of current evidence and theory. | ✓ | 20-21 |

**Note on Item 7 (Sample Size):** The study utilized a convenience sample of all eligible instruments processed over a defined six-month period rather than a formal power calculation to determine sample size.
**Note on Item 9 (Blinding):** Blinding of personnel operating the cleaning devices was not feasible due to the nature of the interventions. Outcome assessors, however, were not involved in the cleaning process.
**Note on Item 12 (Participant Flow):** A flow diagram was not included as all 800 instruments that were included were processed and analyzed, with no dropouts or exclusions from the analysis.

From: Des Jarlais, D. C., Lyles, C., Crepaz, N., & the Trend Group (2004). Improving the reporting quality of nonrandomized evaluations of behavioral and public health interventions: The TREND statement. *American Journal of Public Health, 94*(3), 361-366. For more information, visit: [www.cdc.gov/trendstatement/](http://www.cdc.gov/trendstatement/)
